# Baseline Characteristics, Prognostic Factors, and Treatment Outcomes for Adult Patients With Rhabdomyosarcoma (RMS)

**DOI:** 10.7759/cureus.32961

**Published:** 2022-12-26

**Authors:** Saif ur Rab, Sameen Bin Naeem, Naqib Ullah Baloch, Mussadique Ali Jhatial, Muhammad Waheed, Samir Fasih, Umm-E Kalsoom Awan

**Affiliations:** 1 Medical Oncology, Shaukat Khanum Memorial Cancer Hospital and Research Centre, Lahore, PAK

**Keywords:** spindle cell rhabdomyosarcoma, pleomorphic rhabdomyosarcoma, alveolar rhabdomyosarcoma, embryonal rhabdomyosarcoma, rhabdomyosarcoma (rms)

## Abstract

Background: Rhabdomyosarcoma (RMS) is the most common soft tissue sarcoma of childhood, while in adults it is one of the rarer tumors. Its prognosis is better in children with current treatment modalities; however, it carries poorer prognosis in adults. Recent data on adult RMS is scarce from our part of world. We report outcomes of adult patients with RMS, and with 40 patients; it is the first study to publish such a large data from Pakistan.

Methods: This was a retrospective study that included 64 adult patients aged 18 years and older. After data extraction and scrutiny, a total of 40 patients were segregated with diagnosis of RMS of various varieties who were treated and followed up subsequently. International Business Machines (IBM) Statistical Package for Social Sciences (SPSS), version 26 (IBM Corp., Armonk, NY) was used to evaluate all of the gathered data.

Results: Embryonal RMS (ERMS) was the most common subtype. Factors favoring better overall survival (OS) at 5 years were absence of nodal and distal metastases, treatment with surgery, margin negative resection, and absence of residual disease on postoperative imaging. Adjuvant radiation therapy (XRT) for positive resection margins as well as for residual disease on postoperative imaging also favored better OS at 5 years. Chemotherapy did impart a trend towards better OS; however, it was not significant. Histopathologic subtype and tumor size did not have any significant impact on outcomes. Median progression free survival (PFS) was 11 months and median OS was 15 months.

Conclusions: Adult RMS is a rare disease entity with widely heterogeneous clinical picture and poorer outcomes as compared to the disease of childhood and adolescence. Further prospective studies with larger sample size are required to establish role of patient, disease, and treatment-related factors affecting outcomes in our population.

## Introduction

Rhabdomyosarcomas (RMS) are primitive mesenchymal tumors. They are the most common soft tissue sarcoma of childhood and one of the most common childhood malignancies [1-2]. It is rare in adults, with less than 5% of adults developing RMS [3]. As per classification by WHO in 2020, there are four subtypes: a) embryonal RMS (ERMS), b) alveolar RMS (ARMS), c) pleomorphic RMS (PRMS), and (d) spindle cell / sclerosing RMS (SRMS) [4]. PRMS is currently sub-classified as a high-grade sarcoma composed of undifferentiated round and spindle cells, and it is treated like soft tissue sarcoma [5]. PRMS is the most common RMS subtype in adult patients in contrast to the ERMS and ARMS subtypes in children [6-7]. Prognosis is better in children and 5-year overall survival (OS) in children and adolescents reaches 70% with current treatment modalities [1]. As far as adult population is concerned; prognosis is poorer with 5-year OS between 20% and 40% [8]. A fairly recent report mentioned alveolar subtype of RMS to be the most common variety in adults and that adults present with nodal and distant metastases more frequently as compared to pediatric age group, where prognosis is much better [1, 9-10]. Advanced age, female gender, presence of distant metastases at diagnosis, primary tumor size of more than 5 cm in localized disease all have been associated with poor prognosis in adult patient population [7]. The most common site of adult RMS is head and neck region [11], however; RMS has been reported in rare locations like para-testicular tissues [12-13]. Recent data on adult RMS is scarce from our part of world [14], hence, we report outcomes of adult patients with RMS, and with 40 patients; it is probably the first study to publish such a large data from Pakistan.

## Materials and methods

Objectives

Primary outcomes for this study were progression free survival (PFS) and OS. Secondary outcomes were clinical characteristics and prognostic factors. PFS was defined as duration between diagnosis and date of relapse or last follow up in case patient is in remission. OS was defined as the time from diagnosis to death from any cause or last follow-up.

Methodology

This was a retrospective study that included 64 adult patients aged 18 years and older, with diagnosis of RMS registered to our institute from January 2000 to July 2021. After data extraction and scrutiny, a total of 40 patients were segregated with diagnosis of RMS of various varieties who were treated and followed up subsequently, till November 31st, 2022. Twenty-four patients were excluded on the basis of no treatment or follow up at our institute, either they were registered for investigation purposes or single visit for opinion.

Data collection

Data collected included age, gender, histopathology subtype, size of tumor, presence or absence of nodal or distant metastases at diagnosis, treatment modality, response to treatment, progression or relapse, stage at relapse, treatment at relapse, and status of patient (alive or dead) at last follow up.

Data analysis

Mean and median were calculated for age, while frequencies were determined for gender, histopathologic subtype, tumor size (<5 cm or >5 cm), nodal and distant metastases, treatment modality, response to treatment, progression or relapse, stage at relapse, treatment at relapse and status of patient (alive or dead) at last follow up. PFS and OS were calculated, and curves plotted using Kaplan Meier curves.

The log-rank test was used to compare survival of patient subgroups. IBM SPSS Statistics, version 26 (IBM Corp., Armonk, NY) was used to evaluate all of the gathered data. Patients were included in the study after obtaining an exemption from the institutional review board of Shaukat Khanum Memorial Trust (EX-28-04-20-02).

## Results

Patient characteristics

Median age of our patient population was 24.2 years, with an age range from 18 to 78 years and a male preponderance (Male: 67.5%, n=27, Female: 32.5%, n=13) (Table 1). 

**Table 1:** Patient and disease characteristics.

Patient and disease characteristics		Count	Count%
Gender	Female	13	32.5%
	Male	27	67.5%
Age median, range (24.4 years, 18-78 years)			
Histopathology	Alveolar	4	10.0%
	Embryonal	15	37.5%
	Pleomorphic	9	22.5%
	Spindle cell	5	12.5%
	Unclassifiable	7	17.5%
Tumor size	Less than 5 cm	9	22.5%
	More than 5 cm	31	77.5%
Baseline nodal involvement	No	27	67.5%
	Yes	13	32.5%
Metastasis at presentation	Bone metastasis	3	7.5%
	Lung metastasis	8	20.0%
	No metastasis	29	72.5%
Surgical resection	No	14	35.0%
	Yes	26	65.0%
Extent of resection	Not applicable	14	35.0%
	R0	16	40.0%
	R1	8	20.0%
	R2	2	5.0%
Residual disease post-surgery	Not applicable	14	35.0%
	No	20	50.0%
	Yes	6	15.0%
Chemotherapy	No	6	15.0%
	Yes	34	85.0%
Radiation	No	26	65.0%
	Yes	14	35.0%
Relapse	No	15	37.5%
	Yes	16	40.0%
Progression	Primary	9	22.5%
	Secondary	16	40.0%
Treatment at relapse	No	31	77.5%
	Yes	9	22.5%

Disease characteristics

ERMS was the most common subtype (37.5%, n=15) followed by PRMS (22.5%, n=9), SRMS (12.5%, n=5) and ARMS (10.0%, n=4). However, seven (17.5%) patients were not classified on histopathology. Most common primary location of disease was thigh (20.0%, n=08), followed by testicle and forearm, 17.5% each (n=07), retroperitoneal mass, neck and hip each 5.0% (n=02). Rest of the tumor locations with 2.5% patients (n=01) each were Anal canal, Buttock, Cervix, Lung, Maxilla, Nasal Cavity, Orbit, Pleura, Scapula, uterus, vagina and vocal cord. Majority (77.5%, n=31) presented with tumor size of more than 5 cm, while (22.5%, n=9) had tumor size < 5 cm. At baseline, nodal metastases were present in 32.5% (n=13) and distant metastases in 27.5% (n=11) patients. More patients (20.0%, n=8) presented with lung metastases as compared to those with bone metastases (7.5%, n=3). There were no nodal and distant metastases in (67.5%, n=27) patients at baseline (Table 1). 

Primary treatment

Curative resection was performed for 65% (n=26) patients with majority (40.0%, n=16) achieving R0 resection. Margins were positive for 25.0% (n=10). One third (35.0%, n=14) did not undergo surgery, with most (92.8%, n=13) being metastatic at presentation. Half of the patients (50.0%, n=20) had no residual disease at post-operative follow up imaging, 15.0% (n=6) had residual disease, while residual disease status was not known for rest (35.0%, n=14). Majority of the patients (85.0%, n=34) patients received chemotherapy (adjuvant / palliative) while only one third (35.0%, n=14) were treated with radiotherapy (adjuvant / palliative) (Table 1). Most common chemotherapy regimen was Ifosfamide, Vincristine, Doxorubicin, Dactinomycin (IVA-Do) for 42.5% (n=17) patients followed by Mesna, Adriamycin, Ifosfamide and Dacarbazine (MAID) for 12.5% (n=5) patients. Rest of the 45.0% (n=18) patients received vincristine based different combinations. Patients who received XRT were treated with radical dose in majority 57.14% (n=08) and the rest 42.85% (n=06) received palliative XRT.

Factors affecting outcomes 

Patient Factors

A total of 25 (62.5%) patients progressed with male preponderance (Male: 72.0%, n=18 and Female: 28.0%, n=7). Nine patients had primary progression (Male: 66.6%, n=06 and Female: 33.3%, n=03) and 16 (40.0%) relapsed after some time (Male: 75.0%, n=12 and Female: 25.0%, n=04). 

Disease Factors

**Figure 1:**
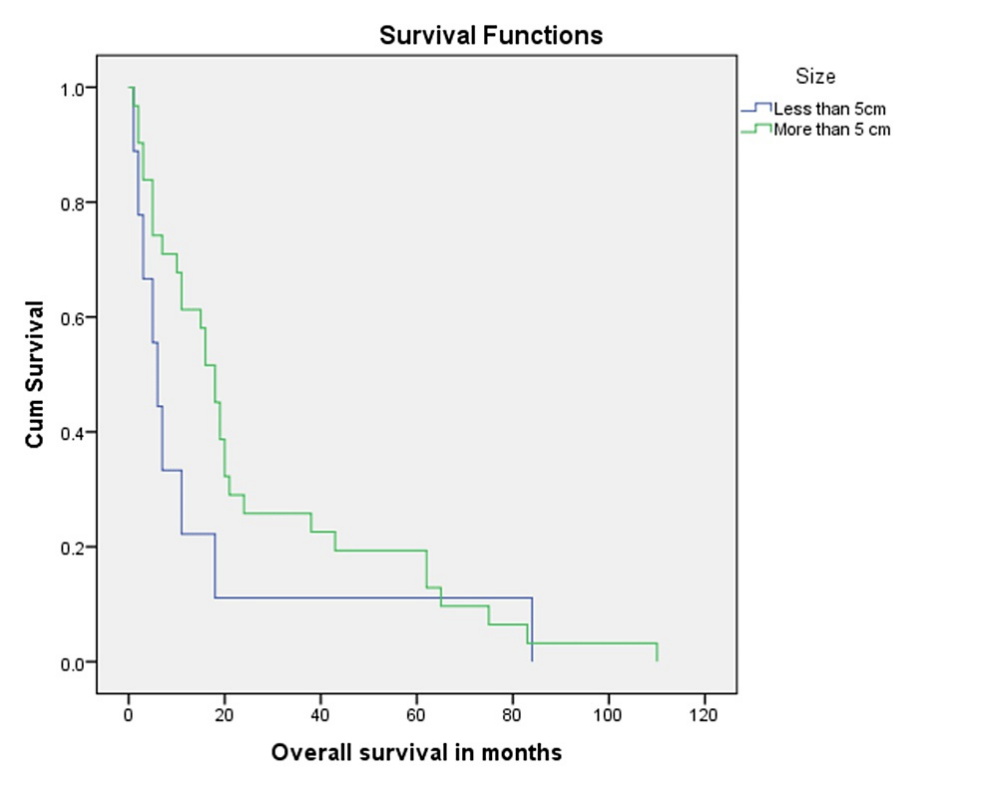
Kaplan Meier curve showing comparison of baseline tumor sizes (p-value = 0.2).

**Figure 2:**
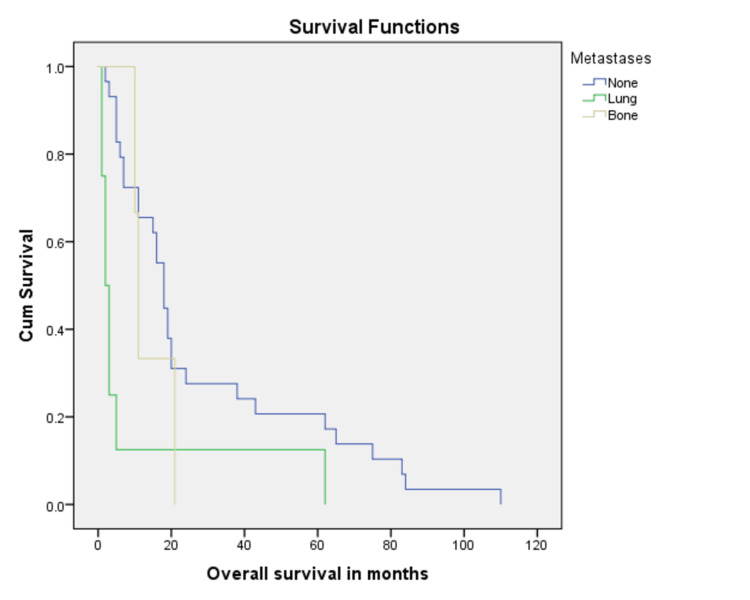
Kaplan Meier curve showing comparison of baseline metastatic sites with non-metastatic disease at presentation (p-value = 0.01).

Most common histopathologic subtype with disease progression or relapse was ERMS (36.0%, n=9) followed by PRMS (20.0%, n=5), however; the impact of histopathologic subtype on prognosis was not statistically significant (p = 0.72). A vast majority of patients with progressive disease had tumor size more than 5 cm at baseline (72.0%, n=18) as compared to those with tumor size less than 5 cm (28.0%, n=7), again not statistically significant (five-year overall survival of 15.2% vs 26.0%, p= 0.21) (Figure 1). Absence of metastatic disease at baseline favored better overall survival with five-year overall survival of 31.5% as compared to 22.2% for those with metastases at diagnoses (Figure 2). 

Treatment Factors

**Figure 3:**
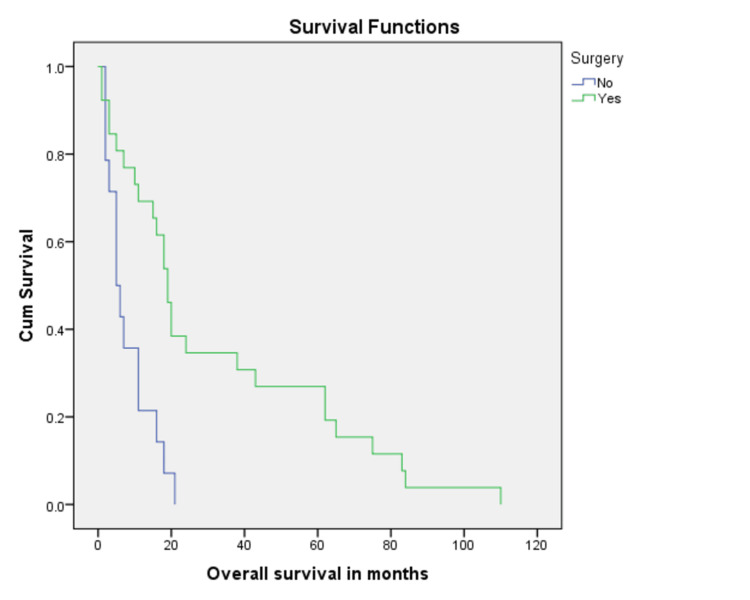
Kaplan Meier curve showing comparison of patients who underwent surgery with irresectable disease (p-value = 0.001).

**Figure 4:**
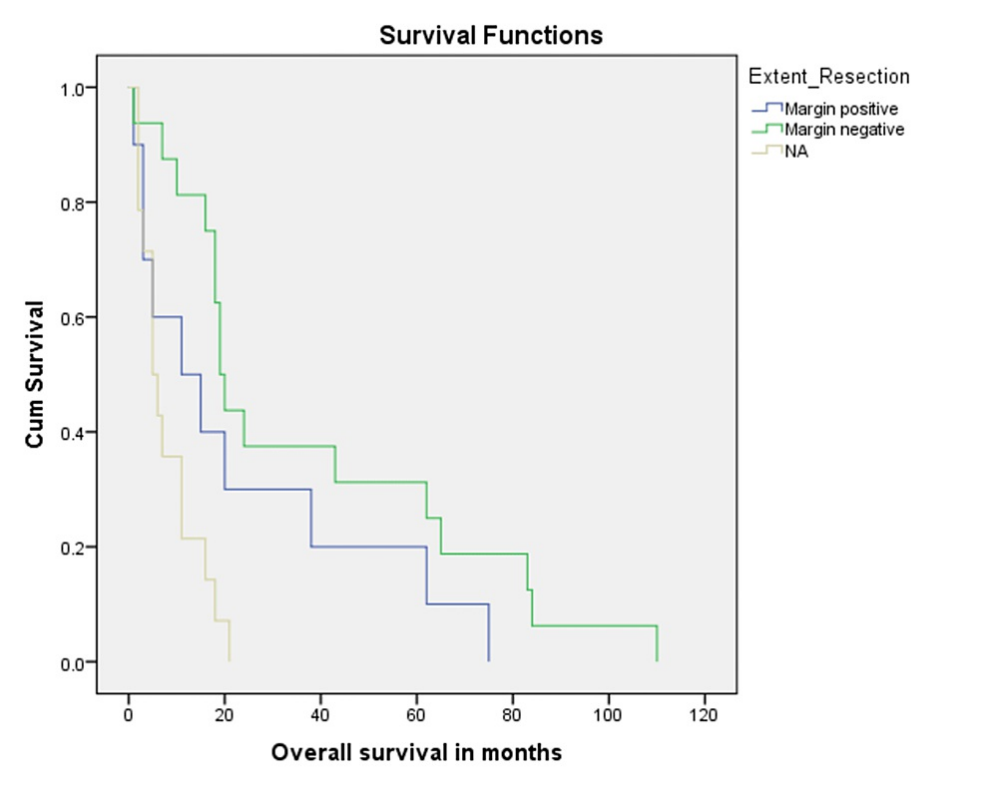
Kaplan Meier curve showing comparison of patients with post-surgical tumor margins (p-value = 0.001).

**Figure 5:**
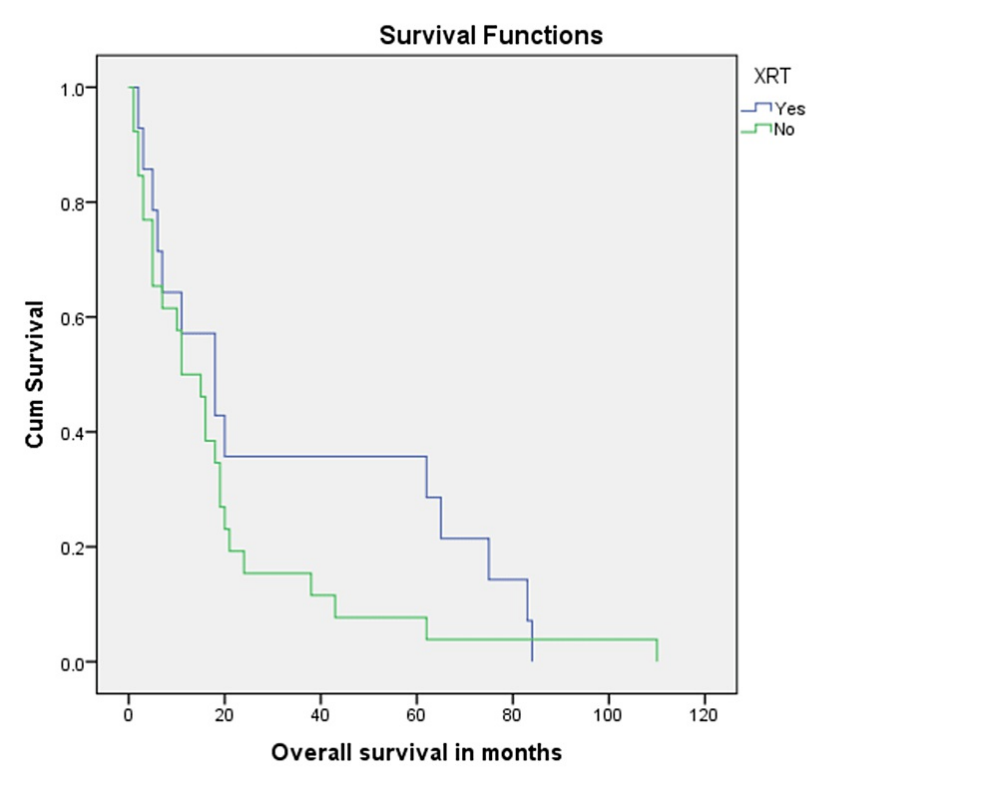
Kaplan Meier curve showing comparison of patients who had radiation therapy with patients who did not have radiation therapy during treatment (p-value = 0.2).

**Figure 6:**
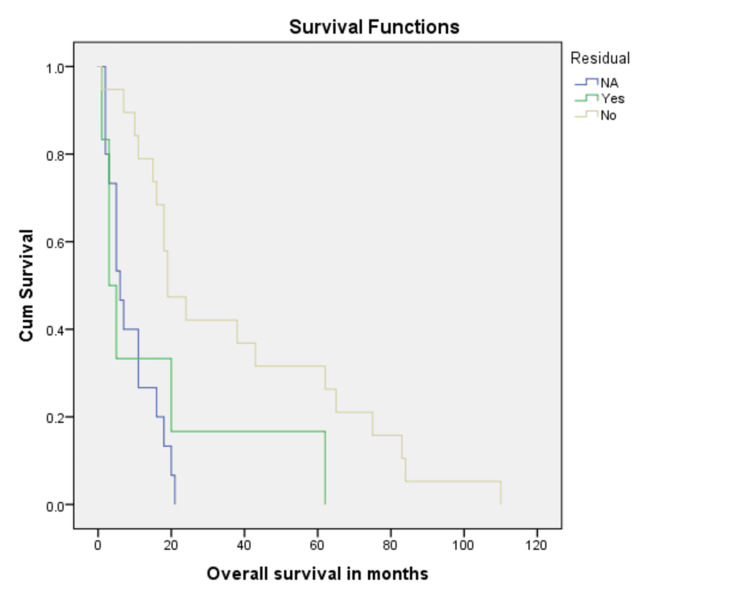
Kaplan Meier curve showing comparison of patients who had residual disease after surgical resection with patient who had complete resection (p-value = 0.001).

Treatment with surgery was associated with better overall survival as compared to no surgical intervention (32.0% vs 8.1%, p = 0.001) (Figure 3). In addition, margin negative resection was associated with better overall survival (37.4%) as compared to 23.3% for those with positive resection margins (Figure 4). When stratified according to adjuvant radiation therapy (XRT), patients who received XRT had better chances to survive as compared to those who did not (62.5% vs 29.0%, p = 0.001) (Figure 5). Residual disease on postoperative imaging was also associated with poor outcomes regarding OS (15.6% vs 37.7%, p = 0.002) (Figure 6). Treatment with chemotherapy (p = 0.06) or radiotherapy (p = 0.8) did not have a significant impact on disease outcomes (Table 2).

**Table 2:** Patient and disease characteristics in comparison with relapse and its corresponding p-value.

Patient and disease characteristics		Relapse			p-value
		No (n)	Primary progression (n)	Yes (n)	
Gender	F	6	3	4	
	M	9	6	12	
Histopathology	Alveolar	2	0	2	0.76
	Embryonal	6	3	6	
	Pleomorphic	4	3	2	
	Spindle cell	2	1	2	
	Unclassifiable	1	2	4	
Size	Less than 5cm	2	2	5	0.2
	More than 5 cm	13	7	11	
Baseline nodal involvement	No	11	5	11	0.4
	Yes	4	4	5	
Metastasis at presentation	Bone metastases	0	0	3	0.02
	Lung metastases	4	4	0	
	No	11	5	13	
Surgery	No	1	6	7	0.04
	Yes	14	3	9	
Extent of resection	NA	1	6	7	0.01
	R0	10	0	6	
	R1	3	2	3	
	R2	1	1	0	
Residual disease in post-operative imaging	NA	1	6	7	0.02
	No	12	1	7	
	Yes	2	2	2	
Chemotherapy	No	3	3	0	0.06
	Yes	12	6	16	
Radiation	No	9	6	11	0.8
	Yes	6	3	5	
Status at last visit	Alive	10	0	3	
	Dead	5	9	13	

Outcomes

**Figure 7:**
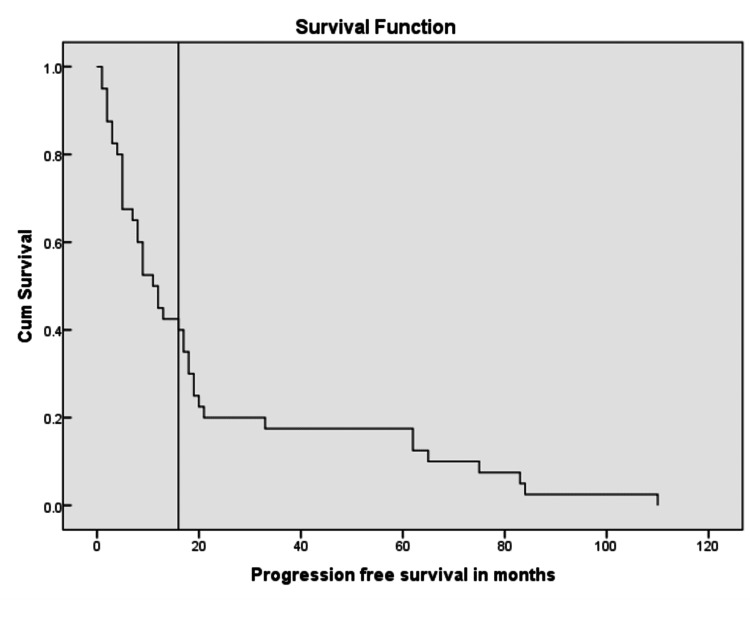
Kaplan Meier curve showing progression free survival in months.

**Figure 8:**
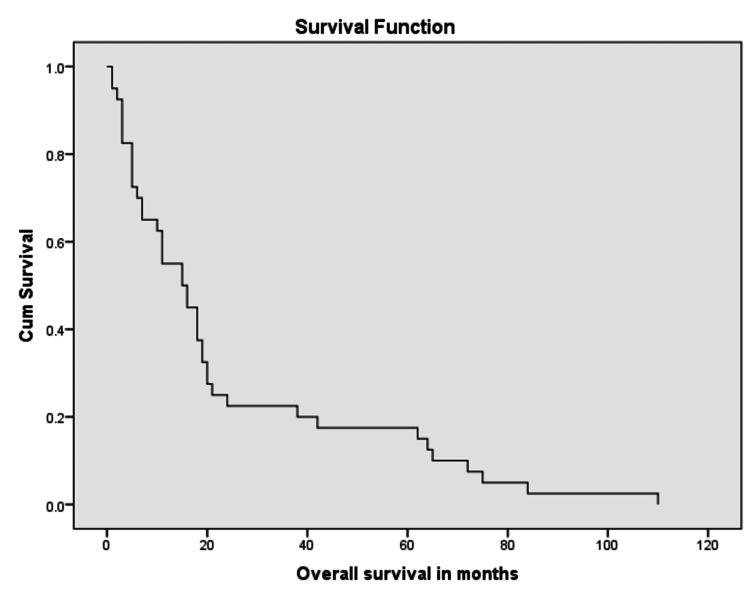
Kaplan Meier curve showing overall survival in months.

**Figure 9:**
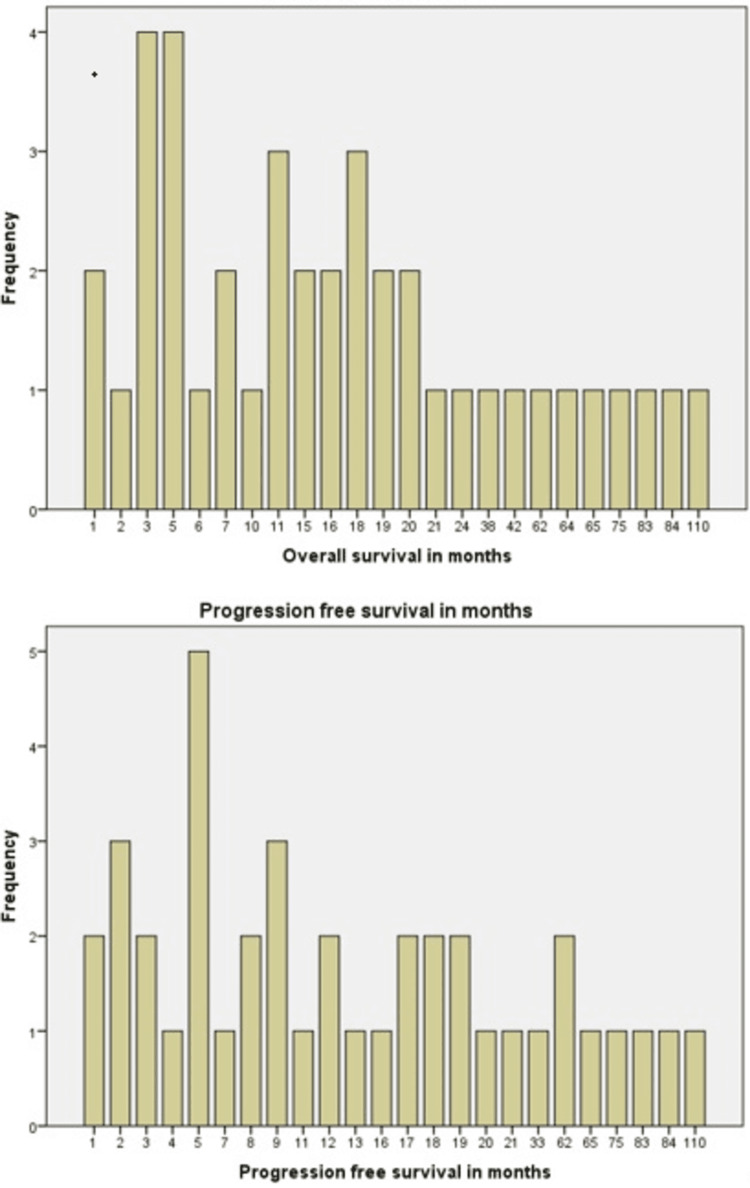
Bar graph showing progression free survival and overall survival for each patient in months.

Primary outcomes for this study were progression free survival (PFS) and overall survival (OS). Median progression free survival was 11 months and median overall survival was 15 months (Figures 7-9).

Patients With Prolonged Survival of More Than 5 Years

Seven patients survived for more than 5 years (ranging from 62 to 110 months), 57.14% (n=04) had ERMS followed by PRMS (28.57%, n=2) and ARMS (14.28%, n=1). Initial tumor size was > 5 cm in most (85.71%, n=06). Five (71.42%) had no metastases at baseline, one each had nodal and distant metastases (14.28%, n=01). All of them underwent surgical excision with margins negative in most (85.71%, n=6) and microscopic positive margin in 14.28% (n=01); however, there was no residual disease in any of them. All except one (85.71%) received chemotherapy and 57.1% (n=04) received XRT. One patient with distant metastases had testicular rhabdomyosarcoma with lung metastases. And the one who did not receive chemotherapy as well as XRT had stage IB uterine PRMS with complete excision.

## Discussion

Rhabdomyosarcoma (RMS) is common in children and adolescents as compared to adults [15]. Because of its rare occurrence in adults, and clinical and biological heterogeneity, it is usually difficult to diagnose [16]. Its relative rarity in adult population and limited data on management of this disease make it necessary that adult patients with RMS be managed by specialist of this disease. The importance of multidisciplinary involvement of medical, surgical and radiation oncologists, radiologists and pathologists is even more in rare malignancies like RMS [17]. As for children, combination therapy implying surgery, chemotherapy, and XRT is utilized for local control as well as to eradicate gross or microscopic metastatic disease [9]. Surgical resection is mainstay of therapy for PRMS, while chemotherapy plays a significant role in case of ERMS and ARMS subtypes [15]. Complete surgical resection is the mainstay of treatment, as reported in our analysis; patients who underwent complete surgical resection had better survival outcomes compared to those with residual disease post operatively. If complete resection is not possible, then control of the primary tumor is imperative with chemotherapy with or without XRT. A report on survival analysis of 553 patients revealed surgery and chemotherapy to have significant impact on outcomes as compared to single treatment modalities or combinations otherwise [18]. Surgery has also been documented to improve outcomes by Esnaola et al. [19]. The same report mentions age, tumor site and tumor size, histopathological subtype, stage of disease and risk group to significantly impact outcomes in addition to treatment modality [18]. Our report mentions primary surgery, tumor margins, and presence of residual disease to significantly impact outcomes. Positive surgical margin has been documented to impact outcomes adversely by Hawkins et al. [7]. However, we report XRT for positive margins, improves OS as compared to those who did not receive XRT. As reported in literature [20], our study concludes that chemotherapy did have a trend toward improved outcomes, however, not significant. Radiotherapy on the other hand, did not improve outcomes, which is in contrast to contemporary reports. The OS of our patient population was lower as compared to published literature (23.6% vs. 52.6%) [18].

## Conclusions

Adult RMS is a rare disease entity with widely heterogeneous clinical picture and poorer outcomes as compared to the disease of childhood and adolescence. Limitations of this study are its retrospective design, small patient population, limited resources for treatment, poor histopathological characterization of around one-fifth of the study population, and last but not the least, improper documentation in patient notes of margin status, residual disease on post-operative imaging. However, this data does form a baseline for further studies outlining baseline characteristics, prognostic factors, and outcomes of this disease. Further prospective studies with larger sample size are required to establish role of patient, disease and treatment-related factors affecting outcomes in our population.
